# Health behaviors and psychological burden of adolescents after parental cancer diagnosis

**DOI:** 10.1038/s41598-022-25256-5

**Published:** 2022-12-05

**Authors:** Kyae Hyung Kim, Min Sun Kim, Seulggie Choi, Sung Min Kim, Sang Min Park

**Affiliations:** 1 Comprehensive Care Clinic, Seoul National University Hospital, Seoul, South Korea; 2 Department of Biomedical Sciences and Family Medicine, Seoul National University Hospital, Seoul National University College of Medicine, 101 Daehak-ro, Jongno-gu, Seoul, South Korea; 3 Department of Pediatrics, Seoul National University Hospital, Seoul, South Korea; 4 Department of Biomedical Sciences, Seoul National University Graduate School, Seoul, South Korea

**Keywords:** Cancer, Health care, Oncology, Risk factors, Human behaviour

## Abstract

This study aims to investigate health behaviors and psychological burdens in adolescent children of cancer parents. We compared health behaviors and mental health outcomes between 266 adolescent children with a parent diagnosed with cancer and 3163 control adolescents aged 12–19 years using data from the Korean National Health and Nutrition Examination Survey (KNHANES) from 2010 to 2018. Alcohol use of adolescents increased between 2 and 5 years after parental cancer diagnosis (adjusted odds ratio [aOR], 1.72; 95% confidence interval [CI], 1.01–2.94) but decreased after 5 years. Parental cancer was associated with increased vaccination uptake in adolescents within 1 year of diagnosis (aOR, 3.19; 95% CI, 1.55–6.54), but after 2 years, there was no difference from rates in their peers. Maternal cancer was associated with increased depression among adolescents (aOR, 1.73; 95% CI, 1.10 − 2.73). Although the risks of suicidal thoughts/plans/attempts increased within 1 year after parental cancer diagnosis (aOR, 2.96; 95% CI, 1.00 − 8.83), it reduced 2 years after diagnosis, leading to no significant difference from the frequency in peers. Within five years after the parent was diagnosed with cancer, support for their adolescent children's health behaviors and mental health is necessary in the community.

## Introduction

Cancer is a leading cause of death worldwide, with nearly 10.0 million deaths in 2020^1^. Cancer development is a complex multi-stage process involving molecular and cellular changes over many years with various risk factors. Accordingly, the incidence of most cancers increases with age, usually more dramatically after middle age^2^. However, a significant proportion of the child-bearing-aged population is still affected. According to the statistical data of the Korean Central Cancer Registry, 10% of male cancer patients and 26% of female cancer patients were in the child-bearing age group between the ages of 30–49 in 2018^3^.

Parental cancer can affect all family members physically, emotionally, socially, and culturally. The patient’s regular routine is disrupted by treatment, potential job loss, and financial problems. Unplanned admission, frequent clinic visits, and decreased function in cancer parents make it difficult for them to care for their children so they can participate in school activities. Higher levels of anxiety and depression in cancer patients are also associated with an increased risk of emotional and behavioral problems and lower quality of life in their children^4–6^. Among children of all ages, adolescents are particularly at risk of psychological problems^7–9^.

Adolescent children show the first signs of puberty in their early teens, approximately between 9 and 13 years^10,11^. They experience a period of dramatic change with rapid physical growth, onset of sexual maturation, and increased self-consciousness and social challenges. Although adolescence is one of the physically healthiest periods in one’s lifetime, there are a notably high proportion of deaths from preventable causes, such as interpersonal violence, suicide, and accidents such as drowning or road traffic accidents^12^. In addition, lifestyle risk factors that dominate life are formed during this period and affect mortality rates later in life. These adolescent health problems are disproportionately influenced by family stability and the social environment^13,14^.

Although the health of children with parental cancer has been studied steadily in Western countries, there is still scarce evidence in Asian cultures. In the present study, we used data from the Korean National Health and Nutrition Survey (KNHANES) to assess the association between factors of parental cancer and health risks of adolescent children after a parental cancer diagnosis.

## Materials and methods

### Study population

The Korea National Health and Nutrition Examination Survey (KNHANES) is a nationwide survey of the health and nutritional status of community-dwelling Koreans. A stratified multi-stage clustered probability design was used to select a representative sample of civilian, non-institutionalized Koreans. The fifth (2010–2012), sixth (2013–2015), and seventh (2016–2018) KNHANES included comprehensive information on the health status, health behavior, and sociodemographic characteristics of the population in 576 national districts. The health interview survey was conducted for all study participants, but the health examination survey and nutritional survey were conducted by randomly selecting about a third of the participants in the health interview survey. The health interview survey in KNHANES was conducted through face-to-face interviews at subjects’ homes by trained interviewers.

**Figure 1:**
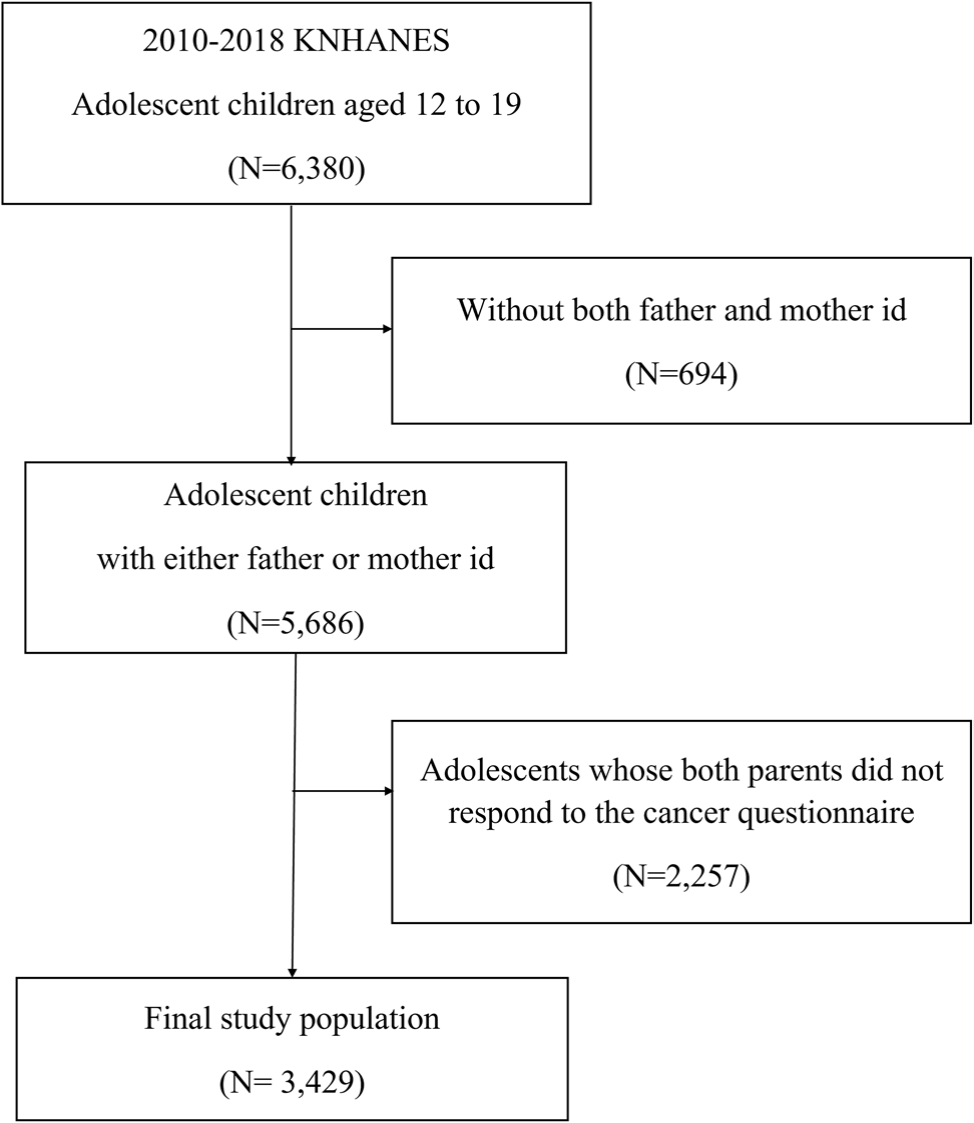
Study population.

When parents and children from one family participated in the survey, the ID of the father and/or mother was coded, and their data were merged for further analysis. The initial participants included 5686 adolescents aged between 12 and 19 years who had the ID code of their parents during the fifth, sixth, and seventh KNHANES surveys, and completed the health interview and health examination surveys. Among them, 2257 participants whose parents did not have a history of cancer were excluded, yielding 3429 adolescents as the final study population (Fig. 1).

### Associated factors

#### Adolescents’ variables

From 2010 to 2018, adolescent participants completed the Health Interview Survey, including age, sex, residential area (rural or urban), and education level. Household income was divided into three levels (< 2000, 2000–5000, or > 5000 thousand won).

Height and weight measurements of adolescent participants were obtained using a standard protocol with an automatic and portable height and weight measuring device. Their weight-for-age was divided into three groups (< 5th, 5th −  < 95th, and ≥ 95th percentile) in reference to the 2017 Korean National Growth Chart^15^.

Adolescents' smoking status was assessed by a question about lifetime ever-smoking experience aged from 12 to 18 years (yes or no) while another question about lifetime smoking measures their lifetime smoking classified into (1) less than 100 cigarettes (2) 100 cigarettes or more (3) never for adolescents aged 19 years or older. For the purpose of analysis, we dichotomously divided them into ever-smoker and nonsmoker. Lifetime alcohol consumption was assessed “Have you ever drank more than one drink in your life?” and the option was yes or no.

The physical activity in the past week was assessed among adolescents using IPAQ (International Physical Activity Questionnaire) between 2010 and 2013, GPAQ (Global physical activity questionnaire) between 2014 and 2016, and hours of moderate or more physical activity between 2017 and 2018. GPAQ reflects leisure-time, worktime, and transport-related PA, allowing accurate evaluation of PA for each area, and the rest of the questionnaires do not distinguish them. Although there was difficulty in simple comparison, we set as a dichotomous variable whether moderate-intensity PA was performed for 150 min or more per week, vigorous-intensity PA more than 75 min, or the equivalent dose of moderate and vigorous physical activity according to the American College of Sports Medicine (ACSM) exercise guideline.

Influenza vaccination during the past 1 year was assessed using a self-reported questionnaire, “Have you been vaccinated against influenza (seasonal flu) in the past year?” And participants who answered “yes” to this question were defined as having received an influenza vaccination. Currently, influenza and Human Papilloma Virus vaccines are recommended in a Korean national vaccination program for adolescents.

Self-rated health consists of the response to the single item, "Would you say your health, in general, is very good, good, fair, poor, or very poor?" We dichotomously categorized “very bad” or “bad” into the “bad participant health” group and “very good,” “good,” or “average” into the “good participant health” group.

Adolescent participants were asked about their stress, “How much stress do you feel in your daily life?” and they rated their stress level as “very high,” “high,” “low,” or “very low.” We dichotomously categorized “very high” or “high” into the “high stress” group and “very low” or “low,” into the “low stress” group^16^.

Depression was screened through a single question, “Over the last 1 year, have you ever had two weeks or more where nearly every day you felt sad or hopeless?” which could be answered yes or no. Adolescents’ suicidal thoughts, plans, and attempts were assessed by asking about the occurrence of those experiences in the last 1 year. An adolescent could answer yes or no to such questions about suicide as “Have you seriously considered suicide in the past year?” “Have you made any concrete plans to commit suicide in the past year?” and “Have you actually attempted suicide in the past year?”.

#### Parental cancer variables

The type of cancer was identified from the medical history section of the questionnaire from the parental survey. From 2010 to 2018, the seven most common types of cancer in Korea were identified through cancer questionnaires (breast, colon, stomach, liver, uterine cervix, lung, and thyroid cancer) and cancer from other sites, including prostate, pancreas, bladder, esophagus, and ovary, among others, was categorized as “other cancer types.” Parental cancer was classified as maternal or paternal. The time after parental cancer diagnosis was calculated by subtracting the age in years at which the participant reportedly was when a parent first had cancer from the age in years at the time of the questionnaire.

### Data analysis

Descriptive statistical methods were used to describe the basic characteristics of the study population, and the results from the chi-squared test for categorical variables and the unpaired t − test for continuous variables are reported. We conducted univariate logistic regression analysis to measure the association between parental cancer and adolescents’ development, tobacco and alcohol use, physical activity, vaccination, subjective health status, stress, depressive mood, and suicidal thought/plan/attempt. To assess the adjusted odds ratios for aforementioned adolescent health behaviors and psychological factors, we used the following covariates in the multivariate logistic regression analysis: sex, age, and household income. A p-value of less than 0.05 was considered significant, and 95% confidence intervals are shown. No weighting was applied. All statistical tests were performed using STATA version 14.0 (Stata Corp., College Station, TX, USA).

### Ethical Approval

This survey complies with the principles of the Helsinki Declaration. The methods were carried out following the relevant guidelines and regulations. All participants agreed to fill out an informed consent form. The KNHANES database is anonymized with the use of its data guided by strict confidentiality guidelines^17^. This present study was approved by the ethics boards of the Seoul National University Hospital (IRB number E-2112–007-1284).

## Results

### Baseline characteristics

Baseline characteristics of the study population are presented in Table 1. The mean age of adolescents with parental cancer was 15.6 ± 2.3 years, which was older than those without parental cancer (15.3 ± 2.3 years). The proportion of elementary school students was higher among adolescents with parental cancer than among adolescents without parental cancer. The difference in sex distribution was not significant. The weight for age for both groups and proportion of smokers and drinkers were not significantly different. Household income and residential area of adolescents also did not differ between the two groups. The proportion of having single parents was 15.8% among adolescents without cancer patients and 11.0% among adolescents with cancer patients.

**Table 1:** Baseline characteristics of adolescent aged from 12 to 19 (n = 3429).

Characteristic	Adolescents without parental cancer	Adolescents with parental cancer	df	Pearson chi-square value	Cramer's V or phi value	*p*^*a*^
	3163 (92.2)	266 (7.8)				
Age (years)	15.3 ± 2.3	15.6 ± 2.3				0.039
**Sex**						
Male	1627 (51.4)	134 (50.4)	1	0.111	0.006	0.739
Female	1536 (48.6)	132 (49.6)				
**Weight for age**						
< 5 percentile	154 (6.5)	12 (5.7)	2	0.329	0.011	0.848
5- < 95 percentile	1,858 (78.8)	168 (80.4)				
≥ 95	346 (14.7)	29 (13.9)				
**Smoking**						
Nonsmoker	2,306 (86.3)	194 (87.0)	1	0.076	−0.005	0.782
Ever-smoker	365 (13.7)	29 (13.0)				
**Alcohol intake**						
Nondrinker	2084 (71.8)	168 (67.5)	1	2.096	0.026	0.148
Drinker	819 (28.2)	81 (32.5)				
**Education level**						
≤ elementary school	688 (17.9)	66 (26.7)	2	3.162	0.032	0.003
Middle school	2,182 (56.8)	126 (51.0)				
≥ High school	970 (25.3)	55 (22.3)				
**Household monthly income**						
< 2000	368 (11.7)	30 (11.3)	2	0.186	0.007	0.911
2,000–5000	1596 (50.5)	138 (51.9)				
> 5000	1196 (37.9)	98 (36.8)				
**Having single parent**						
No	2383 (84.2)	211 (89.0)	1	0.447	−0.036	0.047
Yes	448 (15.8)	26 (11.0)				
**Parental cancer**						
Maternal cancer		201 (75.6)				
Paternal cancer		65 (24.4)				
**Number of parental cancers**						
1		235 (94.8)				
2		13 (5.2)				
**Cancer type of parents**						
Stomach cancer		23 (8.6)				
Liver cancer		4 (1.5)				
Colorectal cancer		13 (4.9)				
Breast cancer		57 (21.4)				
Cervical cancer		29 (10.9)				
Thyroid cancer		97 (36.5)				
Other cancer		48 (18.0)				
**Time since parental cancer diagnosis**						
≤ 1 year		20 (8.1)				
2– < 5 years		95 (38.3)				
≥ 5 years		133 (53.6)				

Data are presented number and percentage (%) or mean ± S.D.

^a^*p* value unpaired t-test for continuous variables or from chi-square test for categorical variables.

Among adolescents with cancer parents, 75.6% had maternal cancer, while 24.4% had paternal cancer. None of the patients had both paternal and maternal cancer. Most cases of parental cancer only involved a single cancer; 5.2% of parents with cancer had a second primary cancer. Among cancer survivors, thyroid cancer was the most common cancer (36.5%), followed by breast (21.4%), uterine cervix (10.9%), stomach (8.6%), colorectal (4.9%), liver (1.5%), and other cancers (18.0%). Among the cancer survivors, ≥ 5 years had elapsed since diagnosis in 53.6% of subjects, 2 −  < 5 years since diagnosis in 38.3%, and ≤ 1 year since diagnosis in 8.1%.

### Adolescent health behaviors

Table 2 shows the health behaviors of adolescents with and without parents with cancer. The proportion of adolescents with abnormal weight for their age was similar between adolescents with and without a parent with cancer. The proportion of current smokers and adolescents who performed physical activity over the recommended levels was similar in adolescents with and without parents with cancer. In an unadjusted model, the proportion of any alcohol use among adolescents whose mother was diagnosed with cancer was higher (odds ratio 1.30, 95% confidence interval [CI], 0.96–1.78); however, the significance was weakened after multivariate adjustment. Interestingly, the adolescents who had any parent with cancer and who had paternal cancer were more likely to have an influenza vaccination than adolescents without parental cancer. However, adolescents with maternal cancer did not show a difference in influenza vaccination rates from that in adolescents without a parent with cancer.

**Table 2:** Behavioral risk factors and psychological factors among adolescent with or without parental cancer.

Characteristic	Adolescents without parental cancer	Adolescents with parental cancer	Adolescents with paternal cancer	Adolescents with maternal cancer
**Abnormal weight for age**^**a**^				
Proportion (%)	20.9	19.5	16.3	20.7
OR	1.00	0.91 (0.64 1.29)	0.72 (0.34 1.48)	1.00 (0.67 1.49)
aOR^b^	1.00	0.83 (0.57 1.20)	0.69 (0.33 1.46)	0.89 (0.59 1.37)
**Ever-smoker, yes**				
Proportion (%)	13.7	11.6	10.9	13.7
OR	1.00	0.94 (0.63 1.42)	0.87 (0.37 2.06)	1.00 (0.64 1.58)
aOR^b^	1.00	0.87 (0.56 1.37)	0.87 (0.34 2.22)	0.90 (0.55 1.47)
**Any alcohol use, yes**				
Proportion (%)	28.2	32.5	28.3	33.9
OR	1.00	1.23 (0.93 1.62)	1.07 (0.60 1.89)	1.30 (0.96 1.78)
aOR^b^	1.00	1.11 (0.78 1.57)	1.08 (0.53 2.23)	1.12 (0.76 1.66)
**Moderate/vigorous intensity physical activity**				
Proportion (%)	67.3	65.3	66.7	64.9
OR	1.00	0.91 (0.66 1.25)	0.95 (0.49 1.82)	0.89 (0.63 1.27)
aOR^b^	1.00	0.95 (0.69 1.30)	0.99 (0.51 1.92)	0.93 (0.65 1.33)
**Influenza vaccination, yes**				
Proportion (%)	26.3	32.4	40.7	29.8
OR	1.00	1.34 (1.00 1.80)*	1.86 (1.07 3.24)*	1.16 (0.83 1.64)
aOR^b^	1.00	1.45 (1.07 1.97)*	1.96 (1.11 3.47)*	1.28 (0.90 1.83)
**Subjective health status (very bad/bad)**				
Proportion	40.0	35.3	28.3	37.6
OR	1.00	0.81 (0.62–1.07)	0.61 (0.35–1.09)	0.91 (0.67–1.23)
aOR^b^	1.00	0.78 (0.59–1.03)	0.60 (0.34–1.07)^†^	0.86 (0.63–1.18)
**Very high/high level stress**				
Proportion	24.9	27.3	21.7	29.1
OR	1.00	1.13 (0.85 1.52)	0.84 (0.45–1.57)	1.27 (0.92 1.76)
aOR ^b^	1.00	1.11 (0.83 1.49)	0.83 (0.44–1.55)	1.24 (0.90 1.72)
**Depressive mood more than two weeks**^**b**^				
Proportion	8.0	10.5	1.75	13.3
OR	1.00	1.35 (0.87 2.09)	0.20 (0.03–1.47)	1.81 (1.15 2.84)*
aOR^b^	1.00	1.29 (0.83 2.01)	0.19 (0.03–1.42)	1.73 (1.10 2.73)*
**Suicidal thought/plan/attempt in the past year**				
Proportion	4.1	5.0	1.75	6.1
OR	1.00	1.25 (0.68 2.30)	0.47 (0.06 3.43)	1.62 (0.86 3.08)
aOR^b^	1.00	1.20 (0.65 2.23)	0.44 (0.06 3.25)	1.58 (0.83 3.01)

Data are presented as percentages (%) and odds ratio (95% CI).

*OR* odds ratio; *CI* confidence interval.

^a^Abnormal weight for age was defined as ≥ 95percentile or < 5percentile of weight for age of Korean adolescent population.

^b^Adjusted for age, sex, and household monthly income.

^c^Recommended physical activity (150 min/week of moderate-intensity physical activity, or 75 min/week of vigorous-intensity physical activity, or the equivalent dose of moderate and vigorous physical activity).

*P < 0.05.

Table 3 shows the health behavior factors according to time since parental cancer diagnosis compared to that in the control group with no parents with cancer. The crude proportion showed that health risk factors, such as abnormal weight for age, smoking, and alcohol use, were highest at 2–5 years after parental cancer diagnosis (23.8%, 18.4%, and 36.2%, respectively). Desirable health behavior such as influenza vaccination were as high 48.5%, immediately after a parental cancer diagnosis at ≤ 1 year and decreased with an increasing number of years after cancer diagnosis to levels similar to those in the control group.

**Table 3:** Behavioral risk factors and psychological factors among adolescent according to time since parental cancer diagnosis.

Characteristic	Adolescents without parental cancer	Adolescents with parental cancer			*P* trend
		Time since parental cancer diagnosis			
		≤ 1 year	2– < 5 years	≥ 5 years	
**Abnormal weight for age**^**a**^					
Proportion (%)	20.9	21.4	23.8	16.4	
OR	1.00	0.99 (0.40 2.47)	1.02 (0.62 1.68)	0.72 (0.40 1.29)	0.416
aOR^b^	1.00	0.91 (0.35 2.32)	0.98 (0.58 1.64)	0.63 (0.34 1.15)	0.306
**Ever-smoker, yes**					
Proportion (%)	13.7	12.9	18.4	7.5	
OR	1.00	1.04 (0.36 2.99)	1.58 (0.93 2.68)*	0.56 (0.28 1.23)	0.137
aOR^b^	1.00	0.95 (0.31 2.90)	1.46 (0.80 2.66)	0.56 (0.25 1.26)	0.209
**Any alcohol use, yes**					
Proportion (%)	28.2	33.3	36.2	28.7	
OR	1.00	1.34 (0.67 2.70)	1.52 (1.01 2.29)*	1.07 (0.70 1.66)	0.397
aOR^b^	1.00	0.98 (0.41 2.34)	1.72 (1.01 2.94)*	0.88 (0.52 1.49)	0.294
**Physical activity above recommendation**^**c**^					
Proportion (%)	67.7	60.7	65.9	66.3	
OR	1.00	0.74 (0.34 1.59)	0.92 (0.58 1.48)	0.94 (0.58 1.51)	0.657
aOR^b^	1.00	0.87 (0.39 1.91)	0.94 (0.58 1.52)	0.98 (0.60 1.60)	0.697
**Influenza vaccination, yes**					
Proportion (%)	26.3	48.5	31.2	28.1	
OR	1.00	2.55 (1.28 5.10)*	1.22 (0.78 1.93)	1.06 (0.67 1.68)	0.056
aOR^b^	1.00	3.19 (1.55 6.54)*	1.33 (0.83 2.11)	1.15 (0.72 1.84)	0.046
**Subjective health status (very bad/bad)**					
Proportion	40.0	31.4	34.3	37.7	
OR	1.00	0.72 (0.35–1.47)	0.82 (0.54–1.47)	0.94 (0.35–1.47)	0.461
aOR^b^	1.00	0.65 (0.31–1.35)	0.80 (0.53–1.22)	0.90 (0.59–1.35)	0.500
**Very high/high level stress**					
Proportion	24.9	22.2	27.6	28.7	
OR	1.00	0.89 (0.40–1.95)	1.18 (0.76 1.83)	1.25 (0.81 1.92)	0.502
aOR^b^	1.00	0.87 (0.39–1.93)	1.20 (0.77 1.86)	1.23 (0.80 1.89)	0.579
**Depressive mood more than two weeks**^**b**^					
Proportion	8.0	5.9	11.0	11.5	
OR	1.00	0.72 (0.17–3.04)	1.43 (0.74–2.73)	1.50 (0.80 2.81)	0.428
aOR^b^	1.00	0.65 (0.15–2.77)	1.42 (0.74–2.72)	1.43 (0.76 2.69)	0.331
**Suicidal thought/plan/attempt in the past year**					
Proportion	4.1	11.8	4.0	3.9	
OR	1.00	3.43 (1.18 10.01)*	1.07 (0.38 2.99)	1.03 (0.37 2.88)	0.138
aOR^b^	1.00	2.96 (1.00 8.83)*	1.04 (0.37 2.91)	0.95 (0.34 2.66)	0.153

Data are presented as percentages (%) and odds ratio (95% CI).

*OR* odds ratio; *CI* confidence interval.

^a^Abnormal weight for age was defined as ≥ 95 percentile or < 5 percentile of weight for age of Korean adolescent population.

^b^Adjusted for age, sex, and household monthly income.

^c^Recommended physical activity (150 min/week of moderate-intensity physical activity, or 75 min/week of vigorous-intensity physical activity, or the equivalent dose of moderate and vigorous physical activity).

*P < 0.05.

In the multivariate logistic model, the proportion of adolescents with abnormal weight for age differed between groups according to time. The smoking rate of adolescents was significantly higher 2 − 5 years after a parental cancer diagnosis with an odds ratio of 1.58 (95% CI, 0.93–2.68), but this association was weakened after multivariate adjustment. Alcohol use was significantly increased 2–5 years after a parental cancer diagnosis with an adjusted odds ratio (aOR) of 1.79 (95% CI, 1.05–3.03). The influenza vaccination rate increased considerably at ≤ 1 year with an aOR of 3.17 (95% CI, 1.54–6.50); however, it decreased 2 or more years after cancer diagnosis to a level that was no different from that in the control group.

### Adolescent mental health

The subjective health status and stress levels did not differ between adolescents with and without parental cancer (Table 2). The multivariate model showed marginal significance that adolescents with paternal cancer were not likely to feel very bad or bad about their own health. Depressive symptoms in adolescents were particularly high when their mothers were diagnosed with cancer (aOR, 1.74; 95% CI, 1.50–2.75). Although suicidal thoughts were more common with a crude proportion of 6.1% vs. 4.1% when mothers had cancer, these associations were non-significant after multivariate adjustment.

Depressive mood was more common 2 years after diagnosis, but this association was also weakened in the multivariate model (Table 3). Approximately 11.8% of adolescents whose parents had cancer had suicidal thoughts/plans/attempts within 1 year after a parental cancer diagnosis. Although the risks of suicidal thoughts/plans/attempts increased within 1 year after parental cancer diagnosis (aOR, 2.96; 95% CI, 1.00 − 8.83), it reduced 2 years after diagnosis, leading to no significant difference from the frequency in peers.

**Figure 2:**
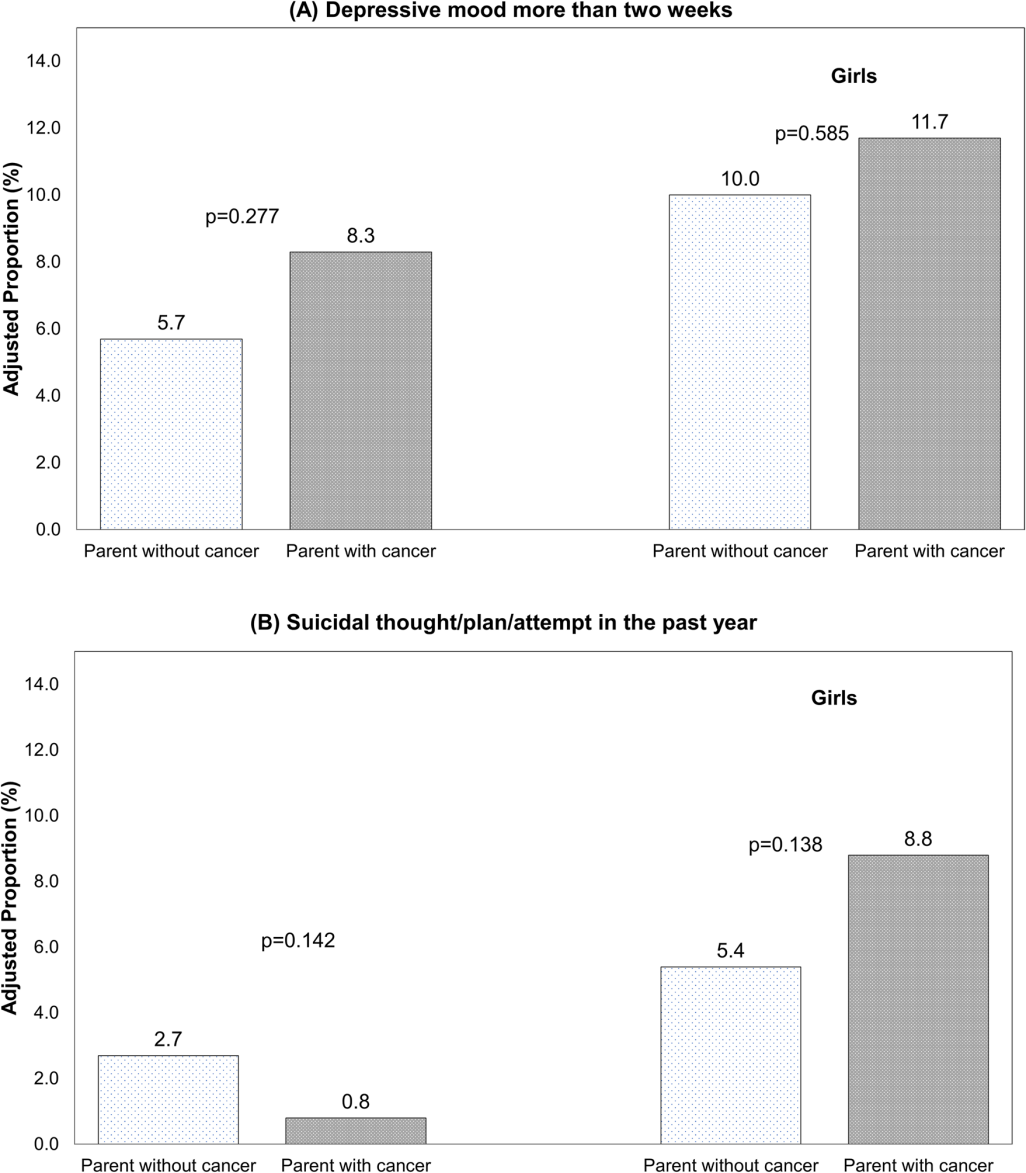
Comparison of depression and suicidal thought among adolescent boys and girls with and without cancer parents. (**A**) Depressive mood more than two weeks. (**B**) Suicidal thought/plan/attempt in the past year.

Depression and suicidal ideation were more prevalent in girls than boys, with or without cancer parents (Fig. 2). In general, the raw proportions increased in boys and girls with cancer parents compared to boys and girls without cancer parents, but there was no statistically significant difference. When the multivariate analysis was conducted, the adjusted proportion of depression was 8.3% for boys with cancer parents and 5.7% for boys without cancer parents, and there was no statistically significant difference (Fig. 2). In addition, girls with cancer parents had a proportion of depression of 11.7%, and girls without cancer patients had a 10.0%, but there was also no significant statistical difference. There was no statistical difference in suicidal thought/plan/attempt between 0.8% of boys with cancer patients' parents and 2.7% of boys without cancer patients. In addition, 8.8% of girls with cancer patients' parents and 5.4% of girls without cancer patients had suicidal thought/plan/attempt in the past year, but there was no statistical difference in the multivariate model (Fig. 2).

## Discussion

Our study shows that, although adolescent children have high behavioral changes and psychological stress in the early stages of their parents' cancer diagnosis, they adapt over time and there is no difference from the general peer group after 5 years in the community setting. Their negative behavior such as alcohol use increased and positive behavior such as vaccination increased for a period of time after a parental cancer diagnosis. There was no significant difference in their subjective health status or stress, but adolescents were more likely to be depressed when their mother was diagnosed with cancer and have suicidal thoughts/plans/attempts immediately after a parent was diagnosed with cancer. However, this vulnerability decreased five years after diagnosis.

Our data showed similar prevalence of adolescent psychiatric problems compared to previous Western studies. Adolescent with parental cancer showed depressive mood in 10.5%, significant stress in 27.3%, and suicidality in 5%. Filial piety, ‘hyo’, is still regarded as an adult children’s duty to care for their old and sick parents in Korea and many Asian countries. Caregiving is strongly aligned with Confucian concept of family and family participation^18,19^. Although studies in other Asian county shows filial piety may lessen caregiver burden^20,21^, it does not mean that the family caregivers do not experience burden or stress^22,23^ In norms of filial piety, there also has been a gender difference for the roles of sons and daughters, and daughters are more likely to serve as caregivers for their parents^21^. As Korean society modernizes, the idea of filial piety is gradually disappearing^24^, and in this study, Korean adolescents with cancer parents show a similar magnitude of risks by gender to those of previous Western studies. ADDIN EN.CITE^25,26^.

Our result corresponds to previous studies that girls reported more psychological symptoms than boys when their parents had a cancer diagnosis^27,28^. In the present study, 8.3% of boys and 11.7% of girls had depressive symptoms. In a Finnish birth cohort study, among 13–17-year-old adolescents, 7.2% of boys and 15.7% of girls with cancer mothers received outpatient psychiatric care^27^. Previous studies suggested that girls are more likely to suffer from distress due to the genetic nature of breast cancer, and girls are likely to have internalizing problems while boys have externalizing problems^6,29^.Stressful life events, such as death, divorce or separation, or unemployment of parents, are known to increase drinking and smoking rates in adolescents^30,31^. Previous studies have suggested that adolescents with family history of cancer have similar health behaviors, such as smoking, alcohol consumption, and nutrition, compared to those without family history of cancer, despite predisposed genetic risks^32,33^. In the present study, the adolescents with parents with cancer did not show differences in their physical development. or health behaviors, compared to those without parental cancer, which is in accordance with previous studies. Their smoking and drinking rates tend to increase at some point after their parents are diagnosed with cancer. Their parents, cancer survivors are recommended to reduce alcohol and smoking cessation to increase their survival, reduce cancer recurrence, and decrease second primary cancer^34,35^. A family-level health behavior intervention program can be considered after a parental cancer diagnosis.

Our study also shows that parental cancer diagnosis can positively influence adolescent children's health behaviors. Previously, there was no evidence of the effect of parental cancer on children's vaccination uptake. However, parental attitudes and social factors are well known to be associated with childhood vaccination^36,37^. Our study showed that the positive influence on vaccination uptake was especially high in adolescents with paternal cancer. It is most likely that not only did the adolescents change vaccination behavior, but their parents also increased their knowledge and had a positive attitude toward vaccination in families caring for fathers with cancer who often had chemotherapy and immunocompromised. Unfortunately, it lasted less than 1 year after the parent’s cancer diagnosis and did not last if the parent survived for more than 5 years. Strategies are needed to ensure that these healthy behaviors are maintained^38^.

Our study corresponds to previous studies that a mother’s illness may have a more substantial influence on adolescents’ emotional well-being than a father’s illness because mothers are more often involved in caring for their children than father’s due to cultural norms^39^. In a previous interview with breast cancer patients revealed that they struggled to be responsible for raising their children while receiving treatment and could not focus on being sick^40^. Moreover, surviving mothers often feel guilty about their children’s abandonment during treatment^40^. This influence is often bidirectional and mutual, and other family members can interact. One study found that children's anxiety and depression increased when their fathers were unable to adjust to bereavement and when families had closed communication about their mother's cancer^41^. The burden on families could be reduced by emphasizing social support for enhancing expression and empathy between sick parents and children.

A previous study reported that the children of cancer patients experienced post-traumatic stress syndrome in the first 4 months of the parental cancer diagnosis^29^. Our study also showed that although they showed a notable increase in suicidal thoughts, plans, or attempts in the first year, they did not show increased suicidality after the second year of a parental cancer diagnosis. Their risky health behaviors increased between 2 and 5 years but decreased from 5 years after parental cancer diagnosis. Although there may be individual differences according to their temperament, stage or unpredictability of parental cancer, or family functioning, the majority of adolescents experience a restoration in psychological and physical function over time^42–44^. Social support may enable them to overcome these crises more easily; adapt to significant physical, cognitive, social, and emotional changes in adolescence^45–47^; and be a catalyst for their posttraumatic growth after negative life events^48,49^.

A major strength of our study was the utilization of nationwide data sources, which enabled us to compare results across the general adolescent population. It is possible to better reflect the reality of families with members with cancer through a community-based design rather than a hospital-centered study. Other strengths include the availability of detailed information on the physical and psychosocial conditions of adolescents.

The significance of this study may serve as reference data for future interventions. Screening for adolescent mental health immediately after the parent's cancer diagnosis is the top priority, and it is also necessary to evaluate their health behaviors. Regular evaluation is required for adolescent use of alcohol and smoking within five years of a parental cancer diagnosis. At the same time, it will be possible to make the crisis act as a positive factor by encouraging physical activity and vaccination of adolescent children. Future research is needed to investigate family-oriented interventions for cancer survivorship to reduce cancer parents' mortality and the long-term risk for their adolescent children.

Some limitations of this study should be considered. First, because this was a cross-sectional study, the causes of differences between cancer survivors and the general population could not be established. Second, several pieces of information were collected from self-reported questionnaires, so reporting bias cannot be excluded. In particular, since the mental health status of adolescents was evaluated through a single question, it was not possible to classify and evaluate the correct psychiatric diagnoses such as major depressive disorder, bipolar disorder, anxiety disorder, affective disorder, or etc. Third, parental cancer stage, previous or ongoing treatment, prognostic factors, and other comorbid chronic diseases were not considered. Fourth, predisposed adolescent history, such as psychiatric diseases other chronic illnesses, school absence, and academic achievement could not be acquired. Fifth, this study mainly investigated cancer survivors, and we cannot generalize our results to all adolescents with a parent with cancer. In addition, we only considered adolescent children of long-term survivors. However, considering that the overall cancer survival rate has improved up to 70% with early detection and treatment, we still need to address the growing problem of cancer survivorship.

## Conclusion

Our study suggests that most adolescent children of cancer survivors did not differ in their smoking, alcohol use, physical activity, and risk of being underweight or overweight compared to general peers. However, alcohol use and vaccination rates somewhat increased immediately after parental diagnosis. These behavioral changes occurred mainly within 5 years of diagnosis, and after 5 years of parental survival, behaviors were the same as those in the general population. There was no significant difference in their subjective health status or stress, but adolescents were likely to become depressed when the mother was diagnosed and have suicidal thoughts/plans/attempts immediately after a parent was diagnosed with cancer. However, this vulnerability decreased from five years after diagnosis. Overall, our study showed that most adolescent children of cancer survivors are resilient to distress associated with parental cancer diagnosis over time. Tailored community-based social support will be required for adolescents to make positive psychological adjustments and grow into adults.

## Data Availability

The data that support the findings of this study are openly available in the official website of KNHANES at https://knhanes.kdca.go.kr/knhanes/sub03/sub03_02_05.do.

## Abbreviations

KNHANES: Korean National Health and Nutrition Survey
ACSM: American College of Sports Medicine
